# Math Anxiety and Its Relations to Arithmetic Fluency and Number Processing: Evidence From Finnish, Finnish‐Swedish, and Swedish Fourth‐Grade Students

**DOI:** 10.1111/sjop.70041

**Published:** 2025-11-12

**Authors:** Pinja Tähti, Jonatan Finell, Anna Tapola, Ellen Sammallahti, Anna Widlund, Bert Jonsson, Riikka Mononen, Johan Korhonen

**Affiliations:** ^1^ Special and Inclusive Education University of Oulu Oulu Finland; ^2^ Faculty of Educational Sciences University of Helsinki Helsinki Finland; ^3^ Department of Applied Educational Science Umeå University Umeå Sweden; ^4^ Faculty of Education and Welfare Studies Åbo Akademi University Turku Finland

**Keywords:** arithmetic fluency, math anxiety, number processing, primary school

## Abstract

The negative relationship between math anxiety and mathematics performance is well established. However, factors such as how math anxiety is operationalized, the specific mathematical domain, gender, and cultural context may influence this relationship. Still, these factors have not been considered together and the results in primary school students have been inconsistent. Thus, this study aimed to investigate how math anxiety is related to arithmetic fluency and number processing in fourth‐grade students across three cultural contexts (Finnish‐ and Swedish‐speaking students from Finland and Swedish‐speaking students from Sweden). In addition, we investigated the dimensionality of math anxiety (i.e., cognitive and affective dimensions) and gender differences in the level of and relations between math anxiety and mathematics performance. The sample included 1022 fourth‐grade students (52.6% girls) from Finland and Sweden. The participants completed a survey measuring their math anxiety and a mathematics performance test (arithmetic fluency and number processing). Confirmatory factor analysis supported a two‐dimensional math anxiety construct for the Finnish‐speaking sample and a unidimensional math anxiety construct for the Swedish‐speaking samples. The negative math anxiety–performance relationship was demonstrated in each sample, showing a slightly stronger association for arithmetic fluency than number processing. On average girls experienced higher levels of math anxiety and boys had better arithmetic fluency. The negative relationship between math anxiety and mathematics performance, especially for number processing, was stronger for boys. The results highlight the relationships between math anxiety and mathematics performance in fourth‐grade students in Finland and Sweden. More research considering cultural (e.g., language) and gender differences is needed.

**Summary**

- The dimensionality of math anxiety and the relationship between math anxiety, arithmetic fluency, and number processing in fourth‐grade students from Finland and Sweden were investigated.
- Confirmatory factor analysis supported a two‐dimensional math anxiety construct with cognitive and affective dimensions for the Finnish‐speaking sample and a unidimensional math anxiety construct for the Swedish‐speaking samples.
- Math anxiety was negatively linked to number processing and arithmetic fluency across samples.
- Gender differences were observed in math anxiety and performance, with girls reporting higher levels of anxiety, and boys outperforming girls in arithmetic fluency.
- The negative relationship between math anxiety and mathematics performance, especially for number processing, was stronger for boys.
- The results highlight the need for more research that considers cultural (e.g., language) and gender differences.

## 1 Introduction

Mathematical skills are important in modern society and are needed in school, work, and everyday life. Students experience different positive (e.g., enjoyment) and negative emotions (e.g., anxiety) while learning mathematics, and these achievement emotions can affect their mathematics performance (Pekrun et al. 2017). Math anxiety has been one of the most investigated negative emotions, and it refers to the feelings of tension and anxiety related to mathematics in different academic or ordinary life situations (Richardson and Suinn 1972). The prevalence of math anxiety is somewhat dependent on age, as younger children in the beginning of their schooling mostly do not experience it (Szczygieł and Pieronkiewicz 2022), whereas about half the adult population has reported moderate levels of math anxiety (Hart and Ganley 2019). Math anxiety has been found to be negatively related to mathematics performance; however until recently, most of the studies have focused on older students (see meta‐analyses; Barroso et al. 2021; Namkung et al. 2019). Studies on primary school students have shown that the relationship between math anxiety and performance may not be significant at the beginning of schooling (e.g., Cargnelutti et al. 2017). However, the relationship stabilizes and becomes significant as students progress into later grades, as evidenced by Pellizzoni et al. (2022) study, where a significant link between math anxiety and performance emerged by early fourth grade. The findings suggest that this phase is critical for the development of math anxiety as the demands of math learning increase and negative experiences have begun to accumulate.

It has been shown that the strength of the math anxiety–performance association can differ due to different factors, such as the operationalizations of math anxiety, mathematical domain, gender, or even cultural factors (Barroso et al. 2021). However, findings concerning primary school students have been inconsistent, often not fully considering these factors. For instance, Wigfield and Meece (1988) identified two math anxiety dimensions: cognitive (i.e., worry component) and affective (i.e., emotional component). Yet, most studies in this age group have focused only on affective (e.g., Caviola et al. 2017; Pellizzoni et al. 2022; Sánchez‐Pérez et al. 2021) or cognitive aspects (e.g., Dowker et al. 2012) separately, or considered math anxiety as a unidimensional construct (e.g., Pekrun et al. 2017). Thus, in this study, we tackled the issue of dimensionality by examining whether the math anxiety, measured by MARS‐E (Henschel and Roick 2017), can be empirically divided into cognitive and affective dimensions already among fourth graders across two language groups, and how these dimensions are related to their mathematics performance. Here, we operationalized mathematics performance as arithmetic fluency and number processing.

The relationship between math anxiety and arithmetic fluency has been more extensively studied in primary school students (e.g., Caviola et al. 2017; Erturan and Jansen 2015; Sánchez‐Pérez et al. 2021) compared to number processing (e.g., Mononen et al. 2022; Szczygieł et al. 2024), although math anxiety has been linked to deficits in low‐level numerical operations, such as symbolic magnitude tasks (Maloney et al. 2011). It is also known that there may be gender differences in both the level of math anxiety and the relations between math anxiety and mathematics performance, at least among older students (e.g., Hembree 1990). However, the findings in primary school students have been inconsistent. Lastly, only a limited number of studies have compared the math anxiety‐performance relationship between different countries or cultural contexts (for an exception, see Ho et al. 2000) although there is some evidence that cultural context, such as educational setting, can affect the level of students' experienced math anxiety and mathematics performance (e.g., Wei et al. 2024). Consequently, this study brings a novel Nordic view with a cross‐country comparison between Finland and Sweden by investigating whether the relationship between math anxiety and mathematics performance differs between fourth‐grade students in these countries, taking into consideration the language and gender aspects. In this study, we include participants from both Finnish and Swedish‐speaking regions of Finland,^1^ as well as Swedish‐speaking individuals from Sweden, which gives the comparison between these three groups an interesting starting point.

### 1.1 Relations Between Math Anxiety and Mathematics Performance

There are different ways to conceptualize and operationalize math anxiety (Henschel and Roick 2020). Wigfield and Meece (1988) suggested that math anxiety could be divided into two dimensions, namely cognitive and affective math anxiety. The cognitive dimension involves worrying about one's performance in mathematics, whereas the affective dimension involves negative emotions, such as nervousness or tension, and even physiological reactions in mathematical situations (Wigfield and Meece 1988). Nevertheless, most studies have treated these dimensions as one (e.g., Pekrun et al. 2017), and only a few studies have separated cognitive and affective dimensions (Harari et al. 2013; Henschel and Roick 2017; Ho et al. 2000).

Prior meta‐analyses have investigated whether the two math anxiety dimensions differ in their relationship to mathematics performance, demonstrating no significant differences between affective and cognitive math anxiety dimensions (Barroso et al. 2021; Namkung et al. 2019). However, the findings concerning third to sixth‐grade students have proven contradictory, as some studies have found the affective factor to be more important (Harari et al. 2013; Ho et al. 2000), while others argue that the cognitive factor is a stronger predictor of math performance (Henschel and Roick 2017). The operationalization of math anxiety can also influence its relationship with performance. For example, Dowker et al. (2016) proposed that studies focusing only on affective math anxiety more frequently report significant relationships compared to those examining only a cognitive dimension. Indeed, most of the studies conducted among students from third to sixth grade have measured math anxiety unidimensionally with items focusing mostly on affective math anxiety. In those studies, a negative relation to mathematics performance has consistently been found (Caviola et al. 2017; Doz et al. 2023; Erturan and Jansen 2015; Justicia‐Galiano et al. 2017; Pellizzoni et al. 2022; Quintero et al. 2022; Sánchez‐Pérez et al. 2021), whereas in a study measuring only cognitive math anxiety, no relation was observed (Dowker et al. 2012).

Although math anxiety has been found to be negatively related to mathematics performance (Barroso et al. 2021; Namkung et al. 2019), the causal ordering of the constructs is still unclear (Carey et al. 2016). There are three theoretical models regarding the math anxiety‐performance relationship (Ma and Xu 2004). The *Deficit Theory* suggests that poor mathematics performance generates higher math anxiety (Carey et al. 2016). For instance, students' prior negative experiences and memories related to mathematics shape students' metacognitive beliefs and attitudes (e.g., self‐efficacy) which, in turn, mediate the effects on math anxiety (Lau et al. 2024). In line with Deficit Theory, studies on students with mathematical learning difficulties have demonstrated that these students experience more math anxiety compared to typically performing students (e.g., Kucian et al. 2018; Passolunghi 2011). On the contrary, the *Debilitating Anxiety Model* suggests that math anxiety hampers the level of mathematics performance (Carey et al. 2016). Math anxiety is thought to affect mathematics performance indirectly through avoidance behaviors (Lau et al. 2024) and cognitive interference, such as taxing working memory resources, which are needed in solving mathematics problems (Finell et al. 2022; Tapola et al. 2025). While some studies have provided support for the Deficit Theory (e.g., Ma and Xu 2004; Sorvo et al. 2019; Wang et al. 2020), others have supported the Debilitating Anxiety Model (e.g., Cargnelutti et al. 2017; Pantoja et al. 2020) making the evidence conflicting. It has also been suggested that the relationship between math anxiety and performance is reciprocal (i.e., the *Reciprocal Model*; Carey et al. 2016) and indeed some findings in primary school‐aged students have supported this (Szczygieł et al. 2024).

In terms of mathematics performance, most of the previous studies in this age group have examined the relationship between math anxiety and arithmetic fluency and found a negative association ranging from weak (*r* = −0.19) to strong (*r* = −0.60) (e.g., Caviola et al. 2017; Erturan and Jansen 2015; Justicia‐Galiano et al. 2017; Sánchez‐Pérez et al. 2021; Schleepen and van Mier 2016; van Mier et al. 2019). However, less is known about the relationship between math anxiety and number processing, although there are some inconsistent findings from both younger students (i.e., first and second‐grade students; Mononen et al. 2022; Szczygieł et al. 2024) and adults (i.e., university students; Maloney et al. 2011; Núñez‐Peña and Suárez‐Pellicioni 2014). Mononen et al. (2022) found that the relationship between math anxiety and number processing was significant in the first grade but not later in the second grade. Similarly, Szczygieł et al. (2024) found that number processing at the beginning of the first grade, but not later in the second grade, predicted math anxiety. There is also evidence suggesting that math anxiety and number processing could be connected later in primary school since Gómez‐Velázquez et al. (2015) showed that math anxiety and number processing were negatively correlated in third‐grade students. Still, the relationship between math anxiety and number processing should be further studied in the later years of primary school.

### 1.2 Gender Differences in Math Anxiety and Performance

Studies with older students and adults often report gender differences, showing females usually experience higher levels of math anxiety (e.g., Devine et al. 2012; Hembree 1990; Stoet et al. 2016), even when gender differences in mathematics performance have not been found (Doz et al. 2023; Goetz et al. 2013). In line with findings from older students, studies on primary school students have demonstrated that girls experience higher levels of math anxiety (Doz et al. 2023; Henschel and Roick 2017; Hill et al. 2016; Sánchez‐Pérez et al. 2021). This has been explained, for instance, by girls holding a higher risk of being predisposed to anxiety in general and in academic contexts (e.g., math anxiety) compared to boys (Carey et al. 2017). Also, different environmental and social factors can contribute to lower competence beliefs among girls (Henschel and Roick 2017). For instance, students may be exposed to gender stereotypic beliefs or expectations (i.e., boys are better in math), which could cause self‐depreciatory thoughts that influence girls' math anxiety (Luttenberger et al. 2018). Nevertheless, numerous studies report no significant gender differences in math anxiety among primary school‐aged students (Dowker et al. 2012; Erturan and Jansen 2015; Quintero et al. 2022; Schleepen and van Mier 2016; van Mier et al. 2019), highlighting the conflicting nature of the results.

Gender differences have also been demonstrated in the relationship between math anxiety and performance. Accordingly, it has been found that the anxiety‐performance relation is stronger or even only significant among girls, although gender differences in the level of math anxiety were not found (Erturan and Jansen 2015; Schleepen and van Mier 2016; van Mier et al. 2019). Currently, evidence regarding gender differences in the math anxiety‐performance link, as well as in the levels of math anxiety experienced by younger students, remains mixed. Hembree's (1990) meta‐analysis suggested that the relationship was stronger among boys, while subsequent meta‐analyses by Ma (1999) and Barroso et al. (2021) found that the math anxiety‐performance relationship was similar for both girls and boys, pointing towards that there are no true gender differences.

Despite mixed findings on gender differences in the math anxiety–performance link, gender differences in mathematics performance are generally minor or negligible among primary school students (see meta‐analyses; Lindberg et al. 2010; Reilly et al. 2015). Regarding gender differences in mathematics performance in Finland and Sweden, the latest results from the Trends in International Mathematics and Science Study (TIMSS) 2019 showed no gender differences in the Finnish fourth‐grade students' mathematics performance, whereas among Swedish students, there were small but significant gender differences favoring boys (Vettenranta et al. 2020). However, some studies have shown some gender differences also in Finland. For instance, Räsänen et al. (2021) found small gender differences in Finnish primary school students' mathematics performance, and interestingly the gender differences were dependent on the mathematics domain. In number processing, girls performed better and the difference increased from grade three to grade nine, whereas in arithmetic fluency boys seemed to perform better than girls (Räsänen et al. 2021).

### 1.3 School Contexts in Finland and Sweden

It is suggested that cultural factors like educational settings and language can influence students' math anxiety, mathematics performance, and the relationships between them (Barroso et al. 2021; Räsänen et al. 2021). To highlight some cultural comparison studies we can turn towards Wei et al. (2024) who observed that third and fourth‐grade students from Italy displayed lower arithmetic skills and higher math anxiety compared to their Chinese counterparts. Similarly, Lichtenfeld et al. (2012) noted that German second and third‐grade students experienced greater mathematics test anxiety than American students, despite the fact that both groups originated from Western countries. Additionally, Räsänen et al. (2021) demonstrated differences in mathematics performance within Finland, where students in the Swedish‐speaking regions outperformed Finnish‐speaking students in number processing and arithmetic fluency. As for the students from Finland and Sweden, neither TIMSS 2019 (Vettenranta et al. 2020) nor PISA 2022 (OECD 2023) assessments found any differences in math performance, even though Finnish students experienced less math anxiety. In addition to the variations in the levels of math anxiety and mathematics performance between countries, differences in the relationship between math anxiety and performance may also be observed. Indeed, Ho et al. (2000) found differences in math anxiety and performance relationships in sixth‐grade students from China, Taiwan, and the United States. While a negative relationship between affective math anxiety and mathematics performance was found in all nations, cognitive math anxiety demonstrated a small positive correlation with math performance only in the Taiwanese sample, perhaps due to cultural differences (Ho et al. 2000). To our knowledge, no prior study has compared the math anxiety‐performance relationship between Finnish and Swedish students.

Cultural variations in math anxiety, mathematics performance, and their relationships, could stem from factors such as different educational systems between countries, but more research on this area is needed (Barroso et al. 2021). Despite many similarities in the educational systems and mathematics teaching between Finland and Sweden, there are some notable differences. In Finland, compulsory education applies to 6–18‐year‐olds, starting with a 1‐year pre‐primary education, and followed by primary education in grades 1–6. In the fourth grade, students have four mathematics lessons per week. The national curriculum (Finnish National Agency for Education 2016), also concerning mathematics, is the same in the Finnish and Swedish‐speaking schools in Finland. Typically, mathematics textbooks are used, which are guided by the national curriculum, as well as other math activities, such as games and using concrete and visual representations. During the school year, students get feedback from their teacher and take some low‐stakes mathematics tests that assess their learning progress. Mathematics is typically taught in primary grades by class teachers, and in upper grades, 7–9, by math subject teachers. If the students need support in their mathematics learning, the three‐tiered support system is followed: general support, intensified support, and special support. Typically, special needs teachers take part in mathematics teaching at the levels of intensified and special support.

In Sweden, the structure of compulsory education follows the one in Finland, and mathematics teaching is guided by the national curriculum (Swedish National Agency for Education 2024). However, some differences do exist. In Sweden, mathematics is typically taught by class teachers in grades 1–6. Though, unlike in Finland, teacher education in Sweden is often subject‐oriented (example mathematics or English), and divided into teachers for grades 1–3 and teachers for grades 4–6. Additionally, the Swedish teacher education program comprises 240 credits, compared to 300 credits in Finland. In grades 7–9, subject‐specialist teachers are responsible for mathematics instruction. Compulsory school starts from the age of 5/6 and ends at 15/16 depending on when in the year the student is born. The Swedish National Agency for Education (2024) stipulates that students in middle school (grades 4–6) must receive at least 410 h of math lessons, amounting to an average of 3.7 h a week. However, it is up to each school to determine how to distribute these hours over the course of middle school. Grades 1–3 must receive at least 420 h of math lessons, whereas the number of hours for high school is 400. Another notable difference between the two countries concerns assessment: in Sweden, mandatory national subject tests (e.g., in mathematics) are given in grades 3, 6, and 9. These are in addition to low‐stakes math assessments and feedback from the teacher during the school year.

### 1.4 Present Study

This cross‐sectional study examines the relationship between math anxiety, arithmetic fluency, and number processing in fourth‐grade students from Finland and Sweden. Fourth grade, when students are typically 10–11 years old in both countries, may represent a critical period for detecting emerging math anxiety and its association with mathematics performance (Pellizzoni et al. 2022). Cultural factors such as educational settings and language can influence students' math anxiety, and mathematics performance (Barroso et al. 2021; Räsänen et al. 2021). Accordingly, this study explores these phenomena within three distinct cultural and language contexts: Finnish, Finnish‐Swedish, and Swedish. We employed a math anxiety measure (MARS‐E) specifically designed to capture the two dimensions of math anxiety. Since Finnish and Swedish are vastly different languages, it is important to validate that the translated measures have the same meaning across language groups and cultures. Thus, the dimensionality will be evaluated separately for each sample, and this evaluation will determine whether using the same statistical model for the three different county‐specific language contexts is possible in further analyses. For mathematics performance, we take into consideration both the lower (i.e., number processing) and higher‐level (i.e., arithmetic fluency) mathematical skills. Our research questions and hypotheses are the following:

How is math anxiety measured with MARS‐E dimensionally structured in fourth‐grade students?

Theoretically, it is justified to expect the two‐dimensional—cognitive and affective—model of math anxiety to fit the data better compared to the unidimensional model of math anxiety (Wigfield and Meece 1988). However, although some empirical studies in this age group have found support for the two‐factor model (Henschel and Roick 2017, 2020), previous results from one of the three research projects included in this study showed that the one‐factor model provided the best fit for the data in Sweden (Finell et al. 2024). Since the present study shares a part of the data used in Finell et al. (2024) study, it is expected that a one‐factor model fits the data best at least in one of the samples (H1).

How is math anxiety related to arithmetic fluency and number processing?

Math anxiety theories suggest that the measured mathematical content may affect the relationship between math anxiety and mathematics performance. For instance, in line with the *Debilitating Anxiety Model*, math anxiety would be more strongly related to complex mathematical content since solving those tasks requires more cognitive resources (Namkung et al. 2019). On the other hand, in line with the *Deficit Theory*, poor number processing may cause math anxiety in mathematical situations (Pellizzoni et al. 2022). Although a prior meta‐analysis by Barroso et al. (2021) did not find the mathematical content to moderate the math anxiety‐performance link, a meta‐analysis by Namkung et al. (2019) showed that more complex mathematical tasks were more strongly related to math anxiety compared to foundational ones. In line with the meta‐analysis by Namkung et al. (2019) and individual studies in this age group, we expect to find a stronger relationship between math anxiety and arithmetic fluency compared to number processing. More precisely, we expect to find a weak to moderate (*r* = −0.19 to −0.29) negative relation between math anxiety and arithmetic fluency (H2.1) as this has been established in previous research (e.g., Caviola et al. 2017; Sánchez‐Pérez et al. 2021). There is only a limited number of studies on the relationship between math anxiety and number processing. A few previous studies also demonstrated a weak to moderate negative relationship between math anxiety and number processing in university (Maloney et al. 2011; Núñez‐Peña and Suárez‐Pellicioni 2014) and first‐grade students (Mononen et al. 2022; Szczygieł et al. 2024). However, the strength of the relationship between math anxiety and number processing has been weaker compared to math anxiety and arithmetic fluency (Mononen et al. 2022). Thus, we expect to find a negative relation between math anxiety and number processing, although weaker compared to arithmetic fluency (H2.2).

Are there gender differences in the mean levels and relations between math anxiety, arithmetic fluency, and number processing?

We expect to find minor or no gender differences in arithmetic fluency and number processing (H3.1) in line with findings from previous meta‐analyses (e.g., Lindberg et al. 2010). However, the evidence for gender differences in math anxiety is not as clear as in mathematics performance. Many studies with older students (e.g., Stoet et al. 2016) and adults (e.g., Hembree 1990) have demonstrated that females experience more math anxiety compared to males. Still, the results for primary school students are inconsistent, and some studies suggest that girls experience more math anxiety (e.g., Doz et al. 2023; Sánchez‐Pérez et al. 2021), whereas some studies have not found any gender differences (e.g., Erturan and Jansen 2015; Quintero et al. 2022). Similarly, gender differences in the relationship between math anxiety and mathematics performance have been mixed. Some individual studies in primary school students have shown a stronger relationship among girls (e.g., Erturan and Jansen 2015); however, meta‐analyses have demonstrated either a stronger relationship for boys (Hembree 1990) or no gender differences (Barroso et al. 2021; Ma 1999). Since the findings from previous individual studies as well as meta‐analyses have been inconsistent, we do not have any specific hypothesis related to gender differences in the level of math anxiety (H3.2) nor in the relation between math anxiety, arithmetic fluency, and number processing (H3.3).

## 2 Methods

### 2.1 Participants

The data for this study was collected as a part of three longitudinal research projects examining the relationship between math anxiety and mathematics performance. Parts of the data have been used in prior studies related to individual projects (e.g., Finell et al. 2024), however, the current study combines three data sets, and therefore the current results represent a substantial contribution on their own. Together 1022 fourth‐grade students (9‐ to 10‐year‐olds, 52.6% girls) from Finland (Finnish‐speaking (FIN) students: iFeelMath‐project; *N* = 332 and Swedish‐speaking (FIN‐SWE) students: SAMSYN‐project; *N* = 295) and Sweden (Swedish‐speaking (SWE) students: Choking under pressure‐project; *N* = 395) were included in this study. All measurements were collected during the spring semester of fourth grade in 2023 and were conducted at schools during regular school hours. The teachers were provided with online measurement instructions and the students performed the measurements individually with computers or tablets. Each measurement was done on separate days during the teacher‐chosen lessons. Each project received Ethical Board consent before the start of the project and informed consents were collected from the students' guardians before the data collection.

### 2.2 Measurements

#### 2.2.1 Math Anxiety

Students' math anxiety was measured with a revised version of the Mathematics Anxiety Rating Scale—Elementary Form (MARS‐E; Henschel and Roick 2017; Richardson and Suinn 1972; Suinn et al. 1988). The revised questionnaire includes 16 items designed to measure cognitive (8 items) and affective math anxiety (8 items). In these items, students rate their emotions related to mathematics lessons, tasks, or tests (e.g., I am worried that I cannot complete my mathematics homework; How nervous are you when you are on your way to mathematics class?). The Likert scale ranged from 1 (not worried/nervous at all) to 4 (very worried/nervous). A Swedish translation of the revised version of MARS‐E (see Finell et al. (2024) for further information) was used in SWE and FIN‐SWE samples in this study. Similarly, a Finnish translation for the MARS‐E was done by the research team and used for this study. The reliability of the MARS‐E subscales was assessed using Cronbach's alpha across three projects. Internal consistency for the Cognitive subscale was high across all projects (*α*_FIN_ = 0.816; α_FIN‐SWE_ = 0.909; *α*_SWE_ = 0.849; *α*_Full sample_ = 0.867). The Affective subscale showed similar reliability (*α*_FIN_ = 0.822; *α*_FIN‐SWE_ = 0.896; *α*_SWE_ = 0.840; *α*_Full sample_ = 0.855). Finally, the use of a General math anxiety factor (all 16 items) demonstrated strong reliability across projects (*α*_FIN_ = 0.880; *α*_FIN‐SWE_ = 0.943; *α*_SWE_ = 0.914; *α*_Full sample_ = 0.916), supporting the internal consistency of the revised MARS‐E in the current study.

#### 2.2.2 Arithmetic Fluency

Arithmetic fluency was measured with a computer‐based Functional Numeracy Assessment—Dyscalculia Battery (FUNA‐DB; Räsänen et al. 2021). FUNA‐DB has been shown to be a reliable and valid tool for measuring arithmetic fluency and number processing in Grades 3–9 (see Hellstrand et al. 2024). For arithmetic fluency, the following tasks were used: number series, single‐digit addition, single‐digit subtraction, and multi‐digit addition and subtraction. In the number series task, there were 36 items together and a maximum of 3 min to solve the problems. The items were presented in order of difficulty. In each item, four numbers were presented, and the students were asked to continue the series according to the rule that the first four numbers formed (e.g., 0 1 2 3 _). The sum score of correct answers was used in the analysis. In the single‐digit addition and subtraction tasks, there were tasks covering addition or subtraction facts with numbers from 1 to 9 (e.g., 2 + 5 = _ and 8–2 = _). Students were asked to calculate as many items as they could during the 2‐min time limit. The calculations were presented in a quasi‐random order. In the multi‐digit addition and subtraction task, items were presented to the students in order of difficulty. The items included two‐ to four‐digit numbers (e.g., 30 + 40 = _, 120–40 = _). The students were asked to answer as fast as possible during the 3‐min time limit. In these three arithmetic fluency tasks, the sum score of correct answers was used in the analysis. As the items were dichotomous, we estimated internal consistency using the KR‐20 coefficient for each subtask. Reliability was high for each subtask and project (*α* ranging 0.86–0.96); see detailed values in Table S1.

#### 2.2.3 Number Processing

Number processing was measured with two tasks from FUNA‐DB: a number comparison (52 items) and a digit dot matching task (42 items; Räsänen et al. 2021). In the number comparison task, two single‐digit numbers were presented, and the students were asked to identify the larger number as quickly as possible. In the digit dot matching task, a single‐digit number (left) and a randomly ordered dot pattern (right) were presented simultaneously. Students were asked to answer if the number and dots were the same or different as fast as possible. Efficiency scores were calculated for number processing by dividing the median reaction time of the correct responses by the percentage of correct responses. Reversed efficiency scores (i.e., a higher score indicates better performance) were used in the analysis for both tasks. The number processing tasks in FUNA‐DB show excellent test–retest reliability both on the latent (*r* = 0.84) and subtask level (*r* > 0.69) (see Hakkarainen et al. 2025).

#### 2.2.4 Data Analysis

The data was analyzed with Mplus (version 8.5) for all latent models. R (version 4.3.0) was used for descriptive analyses. Descriptive statistics were computed for all variables, including means, standard deviations, and correlations. Correlations were estimated for the full sample (Table 1) and separately for each sample and each gender. These are found in the Tables S2–S4. Reliability was assessed through internal consistency measures, using Cronbach's *α* for the math anxiety scale. Reliability statistics were estimated for the full sample and separately for each sample.

**TABLE 1:** Descriptive statistics and correlations.

*N*	*N* (girls/boys)	*M* (SD)	*M* (girls/boys)	SD (girls/boys)		1. MA	2. CMA	3. AMA	4. NP	5. AF
894	467/417	1.609 (0.542)	1.712/1.493	0.552/0.503	1.	—	0.943**/0.942**	0.939**/0.937**	−0.116*/−0.268**	−0.276**/−0.292**
925	482/433	1.566 (0.576)	1.658/1.459	0.598/0.529	2.	0.945**	—	0.778**/0.785**	−0.107*/−0.237**	−0.233**/−0.274**
917	479/427	1.663 (0.583)	1.772/1.537	0.586/0.552	3.	0.944**	0.794**	—	−0.121*/−0.262**	−0.291**/−0.267**
926	487/429	0 (1)	−0.048/0.063	1.043/0.951	4.	−0.208**	−0.184**	−0.209**	—	0.485**/0.512**
882	470/405	0 (1)	−0.153/0.182	0.929/1.054	5.	−0.318**	−0.279**	−0.315**	0.493**	—

*Note:* In the correlation table, the lower triangle presents correlations for the full sample, and the upper triangle for girls/boys.

Abbreviations: AF, arithmetic fluency; AMA, affective math anxiety; CMA, cognitive math anxiety; MA, math anxiety; NP, number processing.

** *p <* 0.001.

* *p* < 0.05.

The dimensionality of the math anxiety construct was analyzed separately for each sample using confirmatory factor analysis (CFA), and each model was evaluated on a set of model fit indices (Hu and Bentler 1999). As the data were on an ordinal scale, the models were estimated using the Weighted Least Squares Means and Variances adjusted (WLSMV) estimator. In the subsequent analyses, math anxiety was used as a multi‐ or unidimensional construct based on the findings from the CFA. Measurement invariance for math anxiety was tested across genders separately for each project using CFA and placing constraints on parameters (to be equal for each gender group). Sample‐based invariance was also assessed if the dimensionality of the math anxiety measure was the same between two samples. Measurement invariance for mathematics performance was tested across gender (see Table S5). For all invariance models: configural, metric, and scalar invariance levels were evaluated, both on their independent model fit, and their relative change from a more liberal to a more conservative state. Configural invariance tests whether the same factor structure holds across groups with parameters freely estimated for both groups. Metric invariance constrains factor loadings to equality, indicating that items contribute equally to the latent factor across groups. Scalar invariance builds on metric invariance by additionally constraining item thresholds (given the ordinal nature of the data), which allows for meaningful comparisons of latent means across groups. Chen (2007) suggests a change in CFI of less than 0.01 and RMSEA of less than 0.015 can be perceived as an acceptable change for holding the models invariant.

Lastly, factor correlations between math anxiety and math performance were estimated, including multiple‐group analysis across gender. These analyses were conducted separately for each sample to understand the potential gender‐specific effects on the associations between math anxiety and each math performance measure across different groups. Wald's test was carried out to determine if the differences in effect sizes were significant. All model fit indices for each latent model were recorded, and can be found in the Table S6.

## 3 Results

### 3.1 The Dimensionality of Math Anxiety

The dimensionality of math anxiety was investigated using CFA separately for each project. The two‐factor model with cognitive and affective math anxiety dimensions was tested and compared to a one‐factor math anxiety model. In the FIN sample (*n* = 319) it was found that the two‐factor model with the cognitive and affective math anxiety (*χ* ^2^ (103) = 412, CFI = 0.915, RMSEA = 0.097, SRMR = 0.083) provided better fit to the data compared to the one‐factor model (*χ* ^2^ (104) = 518, CFI = 0.885, RMSEA = 0.112, SRMR = 0.095). The cognitive and affective factors correlated positively (*r* = 0.77).

In the FIN‐SWE sample (*n* = 250) the two‐factor model provided a good fit for the data (*χ* ^2^ (103) = 279, CFI = 0.971, RMSEA = 0.083, SRMR = 0.045). However, in contrast to the FIN sample, the latent correlations between the cognitive and affective dimensions were very high (*r* = 0.91). Thus, the one‐factor model, which also provided a good fit to the data (*χ* ^2^ (104) = 311, CFI = 0.966, RMSEA = 0.089, SRMR = 0.049) was adopted. Similarly, in the SWE sample (*n* = 382) the one‐factor model was adopted due to the very high latent correlation (*r* = 0.95) between the cognitive and affective dimensions in the two‐factor model. The one‐factor model provided a similar model fit for the data (*χ* ^2^ (104) = 344, CFI = 0.958, RMSEA = 0.078, SRMR = 0.061) compared to the two‐factor model (*χ* ^2^ (103) = 334, CFI = 0.960, RMSEA = 0.077, SRMR = 0.061).

Measurement invariance for gender was assessed in all projects and project invariance in FIN‐SWE and SWE samples since those had the same factor structure for math anxiety, making the comparison between samples possible. The measurement invariance was assessed by comparing the less constrained model (i.e., configural model) to stricter constrained models (i.e., metric and scalar). The measurement invariances are presented in Table 2. The scalar model provided the best fit for the data and the model fit change was acceptable in all models (i.e., change in CFI less than 0.01 and in RMSEA less than 0.015; Chen 2007). These results indicate that the MARS‐E is invariant across genders and projects.

**TABLE 2:** Gender and project invariance for math anxiety.

Gender and project invariance							Model fit change			
Model	*χ* ^2^	DF	*p*	CFI	RMSEA	SRMR	^Δ^ *χ* ^2^	^Δ^CFI	^Δ^RMSEA	^Δ^SRMR
Gender invariance (FIN sample, 2‐factor)										
Configural	485.553	206	0	0.926	0.093	0.089				
Metric	505.824	220	0	0.925	0.091	0.090	20.271	0.001	0.002	0.001
Scalar	510.447	250	0	0.931	0.081	0.091	4.623	0.006	0.01	0.001
Gender invariance (FIN‐SWE sample, 1‐factor)										
Configural	420.478	208	0	0.964	0.092	0.067				
Metric	434.926	223	0	0.964	0.089	0.068	14.448	0	0.003	0.001
Scalar	470.544	254	0	0.963	0.084	0.069	35.618	0.001	0.005	0.001
Gender invariance (SWE sample, 1‐factor)										
Configural	430.163	208	0	0.959	0.075	0.074				
Metric	459.538	223	0	0.956	0.075	0.076	29.375	0.003	0	0.002
Scalar	464.141	250	0	0.960	0.067	0.078	4.603	0.004	0.008	0.002
Project invariance (FIN‐SWE and SWE samples)										
Configural	655.980	208	0	0.962	0.083	0.057				
Metric	724.513	223	0	0.957	0.084	0.059	68.533	0.005	0.001	0.002
Scalar	722.397	254	0	0.960	0.076	0.060	−2.116	0.003	0.008	0.001

### 3.2 The Relationship Between Math Anxiety, Arithmetic Fluency, and Number Processing

**FIGURE 1:**
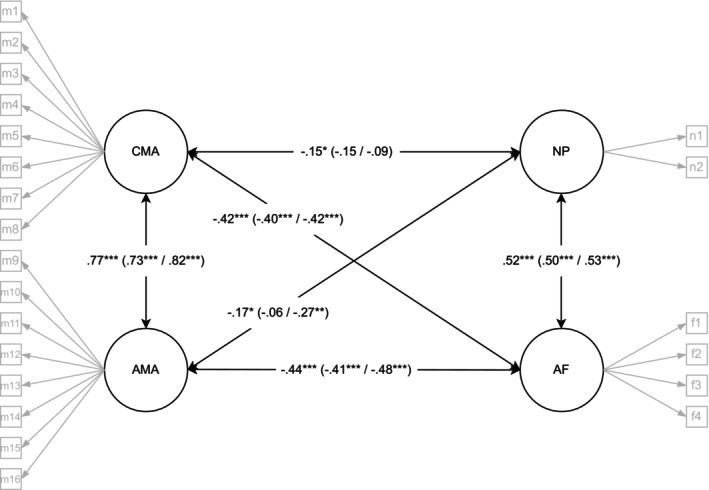
Latent correlations between math anxiety and mathematics performance in the FIN sample. The first correlation presents the total sample, and in parentheses girls/boys correlations. ***p < 0.001, **p < 0.01 and *p < 0.05, AF, arithmetic fluency; AMA, affective math anxiety; CMA, cognitive math anxiety; NP, number processing; CFA with total sample (*n* = 332), *χ* ^2^ (df) = 508 (203), CFI = 0.916, RMSEA = 0.067, SRMR = 0.072; Multigroup CFA with girls (*n* = 180) and boys (*n* = 150), *χ* ^2^2 (df) = 719 (485), CFI = 0.929, RMSEA = 0.059, SRMR = 0.085.

Since the dimensionality of math anxiety differed between the samples, the relationship between math anxiety, arithmetic fluency, and number processing was investigated using CFA separately for each project (see Table S7 for standardized factor loadings). In the FIN sample, math anxiety was used as a two‐dimensional construct with cognitive and affective dimensions. The results showed that both cognitive and affective math anxiety dimensions were negatively related to arithmetic fluency and number processing (Figure 1). As hypothesized, the relationship between cognitive and affective math anxiety was stronger with arithmetic fluency (*r* = −0.42 and *r* = −0.44, respectively) compared to number processing (*r* = −0.15 and *r* = −0.17, respectively). The latent correlations between math anxiety and mathematics performance were similar in magnitude between the two math anxiety dimensions.

**FIGURE 2:**
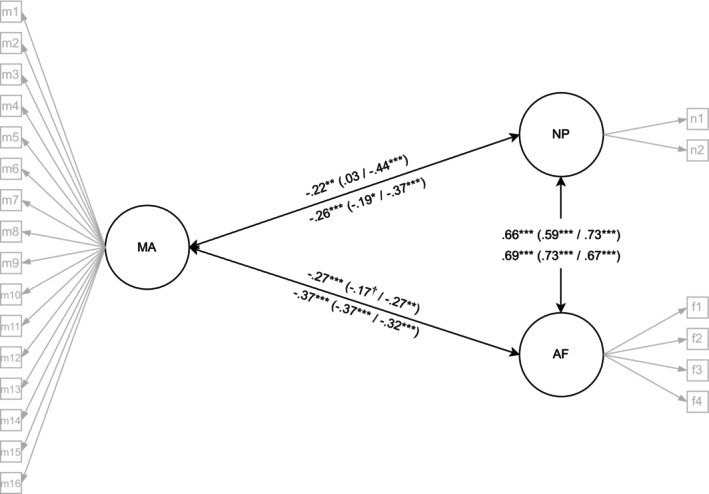
Latent correlations between math anxiety and mathematics performance in the FIN‐SWE (upper) and SWE (lower) sample. The first correlation presents the total sample and in parentheses girls/boys correlations. ***p < 0.001, **p < 0.01, *p < 0.05, and, †*p* < 0.07, AF, arithmetic fluency; MA, math anxiety; NP, number processing; CFA with total FIN‐SWE sample (*n* = 285), *χ* ^2^ (df) = 375 (206), CFI = 0.974, RMSEA = 0.054, SRMR = 0.053; Multigroup CFA by gender (ngirls = 150, nboys = 126), *χ* ^2^ (df) = 675 (466), CFI = 0.965, RMSEA = 0.057, SRMR = 0.079; CFA with total SWE sample (*n* = 395), *χ* ^2^ (df) = 428 (206), CFI = 0.962, RMSEA = 0.052, SRMR = 0.059; Multigroup CFA by gender (ngirls = 194, nboys = 200), *χ* ^2^ (df) = 662 (462), CFI = 0.964, RMSEA = 0.047, SRMR = 0.076.

In the FIN‐SWE and SWE samples, unidimensional math anxiety was used. In line with the findings from the FIN sample, the relationship between math anxiety and mathematics performance was negative in the FIN‐SWE and SWE samples (Figure 2). In both samples the relationship between math anxiety and arithmetic fluency was slightly stronger compared to number processing: in the FIN‐SWE sample, the difference in the relationship between math anxiety and arithmetic fluency (*r* = −0.27) compared to number processing (*r* = −0.22) was not as prominent as in the SWE sample (*r* = −0.37 for arithmetic fluency and *r* = −0.26 for number processing). Both FIN‐SWE and SWE samples demonstrated a slightly lower relationship between math anxiety and arithmetic fluency, but a stronger relation between math anxiety and number processing compared to the FIN sample.

### 3.3 Gender Differences

In the full sample, girls reported higher levels of math anxiety than boys, whereas boys outperformed girls in arithmetic fluency. No gender differences were found in number processing (Table 3). In the FIN sample, girls reported higher levels of cognitive math anxiety, but no difference was observed in affective math anxiety. Boys performed better in both arithmetic fluency and number processing. In both the FIN‐SWE and the SWE sample, girls reported higher levels of math anxiety. While there were no gender differences observed in number processing, boys outperformed girls in arithmetic fluency.

**TABLE 3:** Standardized factor scores and gender differences.

Variable	Mean (SD)	Girl mean (SD)	Boy mean (SD)	*t*‐value	Cohen's *d*
Full sample					
NP	0 (1)	−0.034 (1.011)	0.053 (0.981)	−1.33	−0.09
AR	0 (1)	−0.11 (0.92)	0.136 (1.068)	−3.757***	−0.25
MA	0 (1)	0.226 (0.977)	−0.254 (0.957)	6.18***	0.5
FIN sample					
NP	−0.248 (1.022)	−0.351 (1.087)	−0.108 (0.916)	−2.075*	−0.24
AR	0.097 (0.961)	−0.061 (0.887)	0.294 (1.017)	−3.251**	−0.37
CMA	0 (1)	0.146 (0.926)	−0.176 (1.06)	2.887**	0.33
AMA	0 (1)	0.095 (0.865)	−0.113 (1.126)	1.825^†^	0.21
FIN‐SWE sample					
NP	−0.015 (1.011)	0.012 (0.919)	−0.001 (1.101)	0.103	0.01
AR	−0.126 (0.924)	−0.206 (0.844)	0.013 (0.996)	−1.864^†^	−0.24
MA	0.253 (1.099)	0.554 (0.991)	−0.129 (1.106)	5.05***	0.65
SWE sample					
NP	0.203 (0.931)	0.211 (0.932)	0.195 (0.934)	0.166	0.02
AR	0.006 (1.069)	−0.087 (0.996)	0.097 (1.133)	−1.709	−0.17
MA	−0.166 (0.893)	−0.001 (0.903)	−0.326 (0.854)	3.608***	0.37

*Note:* Under “full sample”, the MA includes cases from FIN‐SWE and SWE samples.

Abbreviations: AF, arithmetic fluency; AMA, affective math anxiety; CMA, cognitive math anxiety; MA, math anxiety; NP, number processing.

*** *p* < 0.001.

** *p* < 0.01.

* *p* < 0.05.

^†^ *p* < 0.07.

Turning to the associations between math anxiety and mathematics performance, some gender‐specific patterns emerged. In the FIN sample, although there were no significant differences in the relationship between affective math anxiety and number processing, the correlation was significant only for boys. From Wald's test we could determine that the only significant differences in effect size could be observed in the correlations between the affective and arithmetic fluency factors (*χ* ^2^ = 1.98, *p* = 0.048). The correlation was stronger for boys. In the FIN‐SWE sample significant gender differences were found in the math anxiety–number processing correlation (*χ* ^2^ = 3.54, *p* < 0.001) but not with arithmetic fluency. The correlation between math anxiety and number processing was stronger for boys. Lastly, no significant gender differences were found in the math anxiety–math performance correlations for the SWE sample.

## 4 Discussion

In this study, we investigated the dimensionality of math anxiety and the relationship between math anxiety, arithmetic fluency, and number processing in fourth‐grade students from Finland and Sweden. We found support for distinct cognitive and affective math anxiety factors in the FIN sample while a unidimensional math anxiety factor fitted the data best in the SWE and FIN‐SWE samples. Math anxiety was negatively related to both arithmetic fluency and number processing across samples. Girls experienced more math anxiety compared to boys and we also found some gender differences in the relationship between math anxiety and mathematics performance across samples.

Our first hypothesis (H1) considering the dimensionality of math anxiety, was partly supported. As theoretically expected, the two‐factor model of math anxiety with cognitive (i.e., worry) and affective (i.e., nervousness) dimensions fitted the data best in the FIN sample. This finding is similar to previous studies investigating the dimensionality of math anxiety measured with MARS‐E among primary school students (Henschel and Roick 2017, 2020; Ho et al. 2000). Also, the high latent correlation between cognitive and affective dimensions (*r* = 0.77) in the FIN sample was in line with the correlations found in previous studies in this age group (Henschel and Roick 2017, 2020). However, the results were somewhat different regarding the Swedish‐speaking samples. Similarly, as in the FIN sample, the two‐factor model provided a good fit for the data, but the latent correlations between the cognitive and affective dimensions were very high, showing that the dimensions could not be meaningfully separated into two dimensions in both Swedish‐speaking samples. Thus, in line with our hypothesis (H1), a unidimensional math anxiety model was adopted for FIN‐SWE and SWE samples. Although speculative, the very high correlation between cognitive and affective dimensions in the FIN‐SWE (*r* = 0.91) and SWE (*r* = 0.95) samples may arise from the Swedish words for worry (i.e., *oroa*) and nervousness (i.e., *nervös*), which may be semantically too close to each other for fourth‐grade students, and therefore the students answer similarly to the questions of both dimensions. Since the same items were used in the Finnish version of the questionnaire and the latent correlation was acceptable (*r* = 0.77), it suggests that the differences stem from the language. Also students' age may influence the observability of math anxiety's dimensionality across different study samples. While research on older students indicates that cognitive and affective math anxiety can be distinguished, studies with early primary school students find these dimensions indistinguishable due to the high correlation between the factors (e.g., Mononen et al. 2022). Although in our study the correlation found in the FIN sample was acceptable, it was higher compared to the ones found in older students (e.g., Ho et al. 2000). Thus, it may be that during this age period, the two dimensions are only starting to become conceptually separated from each other and could become more distinct later on.

In line with the second hypothesis (H2.1), we found a negative relationship between math anxiety and arithmetic fluency in all samples. The strongest relationships were found between cognitive and affective math anxiety and arithmetic fluency in the FIN sample (*r* = −0.42; *r* = −0.44, respectively). The relationship between unidimensional math anxiety and arithmetic fluency was slightly stronger in the SWE sample (*r* = −0.37) compared to the FIN–SWE sample (*r* = −0.27). The strengths of the relations found in this study were in line with the ones that have been demonstrated in previous studies assessing the relationship between unidimensional math anxiety measures and arithmetic fluency (e.g., Caviola et al. 2017; Erturan and Jansen 2015; Justicia‐Galiano et al. 2017; Sánchez‐Pérez et al. 2021). Also in line with our hypothesis, a negative relationship between math anxiety and number processing was found, and the relationships seemed to be slightly weaker compared to arithmetic fluency (H2.2). This finding aligns with the *Debilitating Anxiety Model* and prior results from a meta‐analysis, demonstrating that more complex mathematics tasks, in this case, arithmetic fluency, are more strongly related to math anxiety compared to more foundational tasks such as number processing (Namkung et al. 2019). In our study, the difference between the relationships of arithmetic fluency and number processing with math anxiety was not as prominent in the SWE and FIN–SWE samples (*r*_*FIN‐SWE*_ = −0.22; *r*_*SWE*_ = −0.26) compared to the FIN sample (*r*_*cognitive*_ = −0.15; *r*_*affective*_ = −0.17). The correlation found in the FIN–SWE and SWE samples aligns with Mononen et al. (2022), who reported a negative correlation between unidimensional math anxiety measures and number processing in first‐grade students. Interestingly, while studies like those by Mononen et al. (2022) and Szczygieł et al. (2024) suggest that the relationship between math anxiety and number processing diminishes after the first grade, other research contradicts this trend. For instance, Gómez‐Velázquez et al. (2015) found persistent correlations in third‐grade students, and Maloney et al. (2011) observed similar links even among university students. The finding that number processing is negatively related to math anxiety also in the later grades aligns with the *Deficit Theory*, which suggests that lower number processing ability may cause math anxiety (Barroso et al. 2021). The mixed findings from the relationship between number processing and math anxiety may also be due to the variability in number processing measures. The FUNA‐DB, used in this study, is a validated and reliable tool for assessing number processing from third to ninth grade, showing a linear developmental trend (Hakkarainen et al. 2025; Räsänen et al. 2021). In contrast, some studies may have used measures too familiar or not cognitively demanding enough for older students, potentially masking a relationship between number processing and math anxiety (Mononen et al. 2022; Szczygieł et al. 2024).

There are not many studies that have investigated the relationship between math anxiety and mathematics performance considering both cognitive and affective dimensions (Harari et al. 2013; Henschel and Roick 2017; Ho et al. 2000). Thus, the results of this study provide novel findings for the relationship between cognitive and affective math anxiety and mathematics performance. Whereas some previous studies have shown that only affective math anxiety is related to mathematics performance (Harari et al. 2013; Ho et al. 2000), we found that both cognitive and affective dimensions of math anxiety were negatively related to mathematics performance in the FIN sample. Similar findings have been demonstrated with German fourth‐grade students, where the relationship was slightly stronger for cognitive than affective math anxiety (Henschel and Roick 2017). However, in the present study, we found no difference in the strength of the association. The relatively high correlation between cognitive and affective dimensions may explain why the relationship between mathematics performance and either affective or cognitive math anxiety was similar.

Our third hypothesis (H3.1) considered gender differences in mathematics performance and was partly supported. As hypothesized, we did not find gender differences in number processing at the whole sample level, although a small difference in the FIN sample was found favoring boys. Contrary to our expectations, gender differences in arithmetic fluency at the whole sample level were found, showing that girls had lower arithmetic fluency compared to boys. However, on a sample level, gender differences were significant only in the FIN sample, while differences did not reach the significant level in the FIN‐SWE (*p* = 0.07) and SWE (*p* = 0.09) samples. Previous findings from Finnish fourth‐grade students have shown that girls have lower arithmetic fluency compared to boys (Räsänen et al. 2021). Although this was in line with the results from the FIN sample, this was not the case in the FIN‐SWE sample. The results from the Swedish‐speaking samples were in line with most of the previous studies demonstrating minor or no gender differences in mathematics performance (e.g., Lindberg et al. 2010).

Given the inconsistency in previous findings regarding gender differences in math anxiety, we did not formulate a specific hypothesis for the results (H3.2). The results showed that in all samples, girls experienced higher levels of math anxiety compared to boys. Although the prior results are mixed, there are also studies in this age group (e.g., Doz et al. 2023; Henschel and Roick 2017; Hill et al. 2016; Sánchez‐Pérez et al. 2021) and in older students (e.g., Stoet et al. 2016) that align with our results, demonstrating higher math anxiety for girls. In the FIN sample, girls showed higher levels of cognitive math anxiety, but no gender differences were found in affective math anxiety. This is partly in line with Henschel and Roick (2017) findings, although they found gender differences in both cognitive and affective math anxiety. In their study, gender differences were related to lower competence beliefs in girls (Henschel and Roick 2017). As suggested by previous research, girls' lower competence beliefs in mathematics may be due to environmental or social factors such as exposure to gender‐stereotypic expectations in a home or school environment, and this may cause their higher math anxiety (Luttenberger et al. 2018). Another explanation for girls' higher math anxiety levels is that they may have a stronger predisposition to general anxiety and thus have a greater risk for developing math anxiety as well, compared to boys (Carey et al. 2017).

For the gender differences in the math anxiety–performance relationship, we did not formulate any specific hypothesis due to inconsistent findings from prior research (H3.3). Although it was found that girls reported higher levels of math anxiety, the relationship between math anxiety and mathematics performance was stronger for boys: In all three samples, the relationship between math anxiety and number processing was stronger or even only significant for boys. Similarly, in the FIN–SWE sample, the relationship between math anxiety and arithmetic fluency was stronger for boys. This finding is contrary to the few prior studies demonstrating that the relationship is stronger or only significant for girls (Erturan and Jansen 2015; Schleepen and van Mier 2016; van Mier et al. 2019). Our results indicate that girls may experience higher levels of math anxiety, although they perform well in mathematics. Whereas for boys, math anxiety is more related to lower mathematics performance, especially in the lower‐level skills such as number processing (see Maloney et al. 2011). This finding relates well to the notion by Carey et al. (2017) that girls may be at a greater risk for developing math anxiety through predisposition to general anxiety and not, or at least not only, through lower math performance. Regarding boys, although there may not be as great a risk to develop math anxiety through general anxiety, the development of math anxiety may stem from lower performance in math (Carey et al. 2017), especially from weakness in lower‐level skills such as number processing, as suggested by the *Deficit Theory* (Barroso et al. 2021). However, further research is needed to better understand these aspects, such as the role of general anxiety and other academic anxieties (e.g., test anxiety) or students' motivational self‐beliefs (e.g., self‐efficacy), that may affect the gender differences in the math anxiety–performance link.

### 4.1 Limitations

As this study provides valuable insights, it is not without limitations. First, although our data is rich and representative to answer our research questions, it might not be representative of all fourth‐grade students from Finland and Sweden. As a result, caution is advised on generalizing the results of our cross‐country comparisons. In future studies, it would be beneficial to also include school context factors in the study designs, which might explain the differences between the countries. Second, this study used a cross‐sectional design, which provides a good estimate of the direct correlations between math anxiety and performance. However, as it lacks temporal order, we are indeed limited in discussing the causation of these relationships, as claims about causal relationships would require a longitudinal design. Future research would make gains in investigating the relationship between math anxiety, arithmetic fluency, and number processing with a longitudinal design. Third, the dimensionality of the MARS‐E instrument differed between our samples. Specifically, a two‐factor model fitted the FIN sample, whereas a unidimensional structure fitted the FIN‐SWE and SWE samples better. This difference could stem from linguistic diversity. This discrepancy affects how math anxiety can be interpreted in cross‐cultural comparisons, as the two‐factor model requires distinguishing between cognitive and affective dimensions, while the unidimensional structure relies on a generalized math anxiety. In the future, the instructions for the Swedish version of MARS‐E should be re‐examined, and potential linguistic solutions for the similarities between the words for worry (i.e., *oroa*) and nervousness (i.e., *nervös*) should be investigated. Also, it would be interesting to produce more comparative studies to support the existence of math anxiety dimensions in this age group. Since, in this study, the language of the measurement seemed to impact the identifiability of the two dimensions of math anxiety, more studies with different language groups within the same countries (i.e., in the same school contexts) or same language groups from different countries are needed. The finding that the math anxiety measurement did not result in the same, theory‐driven structure (i.e., two related but conceptually separable dimensions) in the two language groups, suggests that there might be a need to standardize math anxiety measures internationally in order to more reliably compare the results from different countries and cultural contexts.

### 4.2 Conclusions

This study highlights the relationships between math anxiety and mathematics performance in fourth‐grade students in Finland and Sweden, taking into account the language aspect. Our findings confirm a two‐dimensional math anxiety construct for the FIN sample, while the FIN‐SWE and SWE samples demonstrated a unidimensional math anxiety construct. The differences might stem from linguistic or cultural factors, pointing out the importance of considering these factors in cross‐cultural comparisons also in future studies. We observed that math anxiety was negatively linked to number processing (low‐level skills) and arithmetic fluency (higher‐level skills). Gender differences were observed in math anxiety and performance, girls reporting higher levels of anxiety, and boys outperforming girls in arithmetic fluency. The relationship between math anxiety and number processing was stronger or only significant for boys.

## Author Contributions

**Pinja Tähti:** conceptualization, writing – original draft preparation, writing – review and editing. **Jonatan Finell:** formal analysis, investigation, writing – original draft preparation, writing – review and editing. **Anna Tapola:** writing – review and editing. **Ellen Sammallahti:** writing – review and editing. **Anna Widlund:** writing – review and editing, project administration. **Bert Jonsson:** writing – review and editing, funding acquisition, project administration. **Riikka Mononen:** conceptualization, investigation, writing – original draft preparation, writing – review and editing, funding acquisition, project administration. **Johan Korhonen:** conceptualization, investigation, writing – review and editing, funding acquisition, project administration.

## Ethics Statement

The Board for Research Ethics at Åbo Akademi University, Finland, and the Swedish Ethical Review Authority (reference number: 2020‐05982).

## Consent

Written consent from parents.

## Conflicts of Interest

The authors declare no conflicts of interest.

## Supporting information


**Table S1:** KR‐20 coefficients for arithmetic fluency tasks.
**Table S2:** Gender descriptives for SWE sample.
**Table S3:** Gender descriptives for FIN sample.
**Table S4:** Gender descriptives for FIN‐SWE sample.
**Table S5:** Mathematics performance—Gender invariance.
**Table S6:** Confirmatory factor analysis—math anxiety construct without constraints.
**Table S7:** Standardized factor loadings extracted from models in Table S6.

## Data Availability

The data that support the findings of this study are available from the corresponding author upon reasonable request.

## Endnotes

^1^
Finland has two official languages, Finnish and Swedish.
