# Supplementary material for: Math Anxiety and Its Relations to Arithmetic Fluency and Number Processing: Evidence From Finnish, Finnish‐Swedish, and Swedish Fourth‐Grade Students
Source: Scand J Psychol. 2025 Nov 12;67(2):504–18. doi: 10.1111/sjop.70041 (PMC12983999; doi:10.1111/sjop.70041)
Supplement: Supplementary file 1 — Table S1: KR‐20 coefficients for arithmetic fluency tasks. Table S2: Gender descriptives for SWE sample. Table S3: Gender descriptives for FIN sample. Table S4: Gender descriptives for FIN‐SWE sample. Table S5: Mathematics performance—Gender invariance. Table S6: Confirmatory factor analysis—math anxiety construct without constraints. Table S7: Standardized factor loadings extracted from models in Table S6. [file SJOP-67-504-s001.pdf]

Table S1. KR-20 coefficients for arithmetic fluency tasks.

Arithmetic fluency task	SWE sample	FIN sample	FIN-SWE sample
Numbers series	0.863	0.860	0.873
Single-digit addition	0.963	0.964	0.961
Single-digit subtraction	0.963	0.957	0.957
Multi-digit addition and subtraction	0.940	0.932	0.928

Table S2. Gender descriptives for SWE sample.

N	N(girl/boy)	M (SD)	M(girl/boy)	SD(girl/boy)		1. MA	2. CMA	3. AMA	4. NP	5. AF
382	191/190	1.47 (0.46)	1.55/1.38	0.50/0.40	1.	-	0.944**/0.934**	0.954**/0.940**	-0.133/-0.238**	-0.342**/-0.276**
382	191/190	1.42 (0.47)	1.49/1.34	0.52/0.41	2.	0.941**	-	0.810**/0.776**	-0.087/-0.211**	-0.316**/-0.256**
382	191/190	1.52 (0.49)	1.62/1.42	0.53/0.43	3.	0.950**	0.800**	-	-0.146*/-0.249**	-0.327**/-0.268**
376	189/186	0.26 (0.88)	0.26/0.26	0.90/0.86	4.	-0.186**	-0.150**	-0.195**	-	0.625**/0.578**
357	181/175	0.02 (1.08)	-0.12/0.15	1.01/1.13	5.	-0.325**	-0.302**	-0.314**	0.585**	-

***p* < 0.01, **p* <0.05. Lower triangle = full sample, upper triangle = girl/boy

Table S3. Gender descriptives for FIN sample.

N	N(girl/boy)	M (SD)	M(girl/boy)	SD(girl/boy)		1. MA	2. CMA	3. AMA	4. NP	5. AF
285	155/128	1.72 (0.52)	1.77/1.65	0.49/0.53	1.	-	0.898**/0.93**	0.886**/0.954**	-0.132/-0.108	-0.366**/-0.434**
307	165/140	1.65 (0.54)	1.71/1.57	0.55/0.52	2.	0.913**	-	0.606**/0.791**	-0.149†/-0.046	-0.28**/-0.347**
296	162/132	1.77 (0.59)	1.81/1.73	0.56/0.63	3.	0.925**	0.703**	-	-0.084/-0.133	-0.347**/-0.408**
303	168/134	-0.32 (1.05)	-0.43/-0.18	1.13/0.92	4.	-0.140*	-0.123*	-0.118†	-	0.401**/0.384**
290	163/126	0.07 (0.99)	-0.12/0.325	0.91/1.04	5.	-0.414**	-0.324**	-0.389**	0.397**	-

***p* <0.01, **p* <0.05, † <0.07. Lower triangle = full sample, upper triangle = girl/boy

Table S4. Gender descriptives for FIN-SWE sample.

N	N(girl/boy)	M (SD)	M(girl/boy)	SD(girl/boy)		1. MA	2. CMA	3. AMA	4. NP	5. AF
227	121/99	1.72 (0.64)	1.89/1.51	0.63/0.59	1.	-	0.967**/0.939**	0.949**/0.891**	0.105/-0.364**	-0.082/-0.261*
236	126/103	1.70 (0.71)	1.85/1.52	0.70/0.68	2.	0.961**	-	0.844**/0.732**	0.097/-0.377**	-0.039/-0.285**
239	126/105	1.76 (0.65)	1.96/1.51	0.64/0.59	3.	0.940**	0.827**	-	0.011/-0.298**	-0.171/-0.195
247	130/109	0.0 (1)	-0.01/0.03	0.96/1.07	4.	-0.148*	-0.145*	-0.171*	-	0.468**/0.664**
235	126/104	-0.11 (0.88)	-0.25/0.06	0.83/0.92	5.	-0.247**	-0.212**	-0.274**	0.56**	-

***p* < 0.01, **p* <0.05. Lower triangle = full sample, upper triangle = girl/boy

Table S5. Mathematics performance - Gender invariance.

	Gender Invariance						Model Fit Change			
Model	χ^2	DF	*p*-Value	CFI	RMSEA	SRMR	$^{\Delta}\chi 2$	$^{\Delta}$CFI	$^{\Delta}$RMSEA	$^{\Delta}$SRMR
Full sample										
Configural	64.782	16	< 0.001	0.986	0.081	0.020				
Metric	66.253	20	< 0.001	0.987	0.07	0.021	1.471	0.001	0.011	0.001
Scalar	105.506	24	< 0.001	0.976	0.085	0.032	39.253	0.011	0.015	0.011
FIN sample										
Configural	45.443	16	< 0.001	0.971	0.11	0.033				
Metric	46.831	20	< 0.001	0.974	0.094	0.036	1.388	0.003	0.016	0.003
Scalar	59.599	24	< 0.001	0.965	0.099	0.046	12.768	0.009	0.005	0.01
FIN-SWE sample										
Configural	35.585	16	0.003	0.98	0.1	0.024				
Metric	36.295	20	0.014	0.984	0.082	0.026	0.71	0.004	0.018	0.002
Scalar	63.847	24	< 0.001	0.96	0.116	0.046	27.552	0.024	0.034	0.02
SWE sample										
Configural	31.209	16	0.013	0.99	0.07	0.021				
Metric	34.371	20	0.024	0.991	0.061	0.026	3.162	0.001	0.009	0.005
Scalar	44.406	24	0.007	0.987	0.066	0.033	10.035	0.004	0.005	0.007

Note: Math performance consisted of both number processing and arithmetic fluency factor.

Table S6. Confirmatory factor analysis - math anxiety construct without constraints

Project	χ^2	DF	*p*-value	CFI	RMSEA	SRMR	N	Correlation
SWE sample (1F)	344.279	104	<0.001	0.958	0.078	0.061	382	-
FIN sample (2F)	411.841	103	<0.001	0.915	0.097	0.083	319	0.77
FIN-SWE sample (1F)	310.585	104	<0.001	0.966	0.089	0.049	250	-

1F = One-factor model, 2F = Two-factor model.

Table S7. Standardized factor loadings extracted from models in Table S6.

Item	FIN sample	FIN-SWE sample	SWE sample
CMA1	0.772	0.831	0.847
CMA2	0.817	0.818	0.769
CMA3	0.757	0.885	0.876
CMA4	0.715	0.779	0.681
CMA5	0.617	0.722	0.665
CMA6	0.592	0.826	0.66
CMA7	0.582	0.876	0.791
CMA8	0.699	0.76	0.785
AMA1	0.715	0.781	0.734
AMA2	0.794	0.836	0.811
AMA3	0.817	0.829	0.801
AMA4	0.744	0.86	0.788
AMA5	0.653	0.776	0.742
AMA6	0.623	0.802	0.73
AMA7	0.674	0.824	0.679
AMA8	0.598	0.771	0.68

Note. CMA = Cognitive math anxiety, AMA = Affective math anxiety,
FIN sample treats CMA and AMA items as separate constructs.
